# The real-time status of strong and weak islands

**DOI:** 10.1371/journal.pone.0263879

**Published:** 2022-02-11

**Authors:** Derya Cokal, Patrick Sturt

**Affiliations:** 1 University of Cologne, Department of German Language and Literature I–Linguistics, Cologne, Germany; 2 Department of Psychology, The University of Edinburgh, Edinburgh, United Kingdom; University of California, San Diego, UNITED STATES

## Abstract

In two eye-tracking reading experiments, we used a variant of the filled gap technique to investigate how strong and weak islands are processed on a moment-to-moment basis during comprehension. Experiment 1 provided a conceptual replication of previous studies showing that real time processing is sensitive to strong islands. In the absence of an island, readers experienced processing difficulty when a pronoun appeared in a position of a predicted gap, but this difficulty was absent when the pronoun appeared inside a strong island. Experiment 2 showed an analogous effect for weak islands: a processing cost was seen for a pronoun in the position of a predicted gap in a *that*-complement clause, but this cost was absent in a matched *whether* clause, which constitutes a weak island configuration. Overall, our results are compatible with the claim that active dependency formation is suspended, or reduced, in both weak and strong island structures.

## Introduction

Sentences may include dependencies between non-adjacent words or phrases. For example, in a sentence (A.a) below, *the magazine* is not adjacent to the preposition *about*, but instead there is a *gap* (marked “___”) where *the magazine* would normally appear in a canonical sentence like *The hairdresser talked about the magazine*.

A.a. This is **the magazine** that Jane said [that the hairdresser had talked about __].

Such *unbounded dependencies* are subject to *island constraints* [1], which prohibit—or limit the acceptability of—dependency links across certain types of domain boundaries. Specifically, the acceptability of a dependency between two linguistic elements, where one element is inside a phrase or clause X, and the other is outside X, is affected by characteristics of X, or how X is syntactically linked with the surrounding phrases. For example, consider the unbounded dependency in A.a as well as those in A.b-d (note that unbounded dependencies relevant to islands include not only relative clauses such as “the magazine that you talked about __” but also wh-questions, such as “Which magazine did you talk about __?”):

A.b. *This is **the magazine** that Jane wondered [whether the hairdresser had talked about **__**].
A.c *Jane liked **the magazine** that I met the hairdresser [who had talked about **__**].
A.d. *Jane liked **the magazine** that [the editor of **__**] had been seen at the salon.

In each of the sentences in A.a-d, there is a dependency between *the magazine* and a “gap” position, marked with an underscore later in the sentence, and in each case, the gap is inside a domain marked by square brackets, while *the magazine* is outside this domain (more specifically, the dependency is between a relative operator co-indexed with “the magazine” and the gap). However, intuitively, these dependencies lead to different degrees of acceptability, with the starred examples A.b-d being degraded relative to A.a. This is because the domains demarcated in square brackets in A.b-d are *islands* with respect to unbounded dependencies, while the relevant domain in A.a is not.

Traditionally, a distinction has been drawn between weak and strong islands [2]. In terms of intuitive acceptability, strong and weak islands are distinguished in at least two ways. The first way in which they are distinguished is that strong islands give rise to a greater sense of unacceptability than weak islands. There is some experimental evidence for this: although strong and weak islands are not usually directly compared in acceptability judgment experiments, violations of strong islands tend to receive lower mean acceptability ratings than violations of weak islands [3, 4]. Another way in which strong and weak islands are claimed to differ is that the intuitive acceptability of weak islands depends on the extracted element. For example, arguments can be extracted from weak islands in some circumstances, but not adjuncts [5]. As another example, the complexity of the extracted phrase can affect the acceptability of extraction from weak islands. For example, the weak island sentence *Who do you wonder whether John likes ___*, where the extracted element is the simple wh-phrase *who*, may be judged to be less acceptable than *Which boy do you wonder whether John likes __*?, where the extracted element is the complex wh-phrase *which boy* (see [2] for further discussion and examples). In contrast, it is often claimed that strong islands are not malleable in the same way (though see [6] for experimental evidence indicating that the situation may not be as simple as this).

Strong islands can be identified in terms of the position that the island domain occupies in the structure of the sentence, for example, by virtue of being an adjunct (A.c), or a subject (A.d). However, this is not generally the case for weak islands. For example, the structural position of the weak island in (A.b) is effectively identical to that of the analogous constituent in (A.a), which is not an island at all; both are tensed clausal complements of their respective verbs. What distinguishes (A.b) is the fact that the clause is interrogative, and is introduced by the *wh-*complementizer *whether*. Here, we should add that, arguably, the island in 1b A.b is due to the presence of a wh-operator in the interrogative clause, which intervenes between the relative operator coindexed with *the magazine* and its trace. However, this relates to internal properties of the clause, rather than the way in which it is attached into the sentence structure. This contrasts with the analogous clause in (A.a), which is declarative, and is introduced by the declarative complementizer *that*. Thus, while strong islands are islands by virtue of structural position (for example, being a subject, or an adjunct within the structure of the sentence), weak islands are arguably islands by virtue of their features (for example, bearing the “wh” feature marked by the complementizer *whether*). The purpose of the present paper is to examine how strong and weak islands are processed in real time, and we will return to discuss how this might be impacted by the differences between the two types of islands in the discussion below.

Sentence comprehension is essentially an incremental process, and to a certain approximation, the parser incorporates each new word into the developing interpretation. Many real-time psycholinguistic studies have examined filler-gap dependencies (i.e., unbounded dependencies) [7–16], and the consensus of these studies is that, in the absence of island constraints, the processing of such dependencies is highly incremental. In particular, there is evidence that the processing of filler-gap dependencies is not only incremental, but also *predictive*, in that comprehenders use an Active Filler Strategy [15]. According to this strategy, once the first element of an unbounded dependency (i.e. a filler) has been processed in the input, the parser actively expects a gap to occur in the closest grammatical position [16]. One of the main sources of evidence for this strategy is the filled gap effect, which can be illustrated by considering example B, from a study by Stowe [11]:

(B) My brother wanted to know **who** Ruth will bring **us** home to __ at Christmas.
(C) My brother wanted to know if Ruth will bring **us** home to Mom at Christmas.

According to the *Active Filler Strategy*, once the parser has processed the filler *who* in (B), a gap is actively expected in the input. The reader initially predicts a gap immediately after the embedded verb (i.e., bring), thus forming a dependency with the filler **who**. However, when the overt direct object (i.e., **us**) is received in the input, this is incompatible with the initial expectation, leading to processing disruption in (B). In contrast, example (C) contains an *if-*clause, and therefore does not include an unbounded dependency, so no gap is expected, leading to the prediction of less processing difficulty in (C) relative to (B), at or following the word “us”. This overall pattern, which is known as the *filled gap effect*, was found in a self-paced reading study reported by Stowe [11], providing evidence for the Active Filler Strategy.

Given the incremental nature of filler-gap processing, one important question is whether island constraints are applied in real time; in other words, whether the processor is immediately sensitive to island domains at the relevant point in processing the string, or alternatively, whether island constraints may be temporarily ignored during the processing of a filler-gap sentence. The latter possibility was suggested by [17], on the basis that, according to certain syntactic theories, island-violations can be generated by the grammar at some level of representation, but are later filtered out during the derivation [18]. However, evidence suggests that island constraints do indeed modulate sentence processing in real time, at least for strong islands. For example, Stowe’s filler-gap study [11] did not show evidence for an increase in reading disruption for the noun phrase “Greg’s older brother”, which appears as the complement of the preposition “about” in the subject island in (D), relative to the if-clause control in (E). This suggested that readers do not predictively form the filler-gap dependency in this strong island sentence structure, and thus that the processor is immediately sensitive to this type of island information at the relevant point during the processing of the sentence.

(D) The teacher asked what [the silly story about **Greg’s** older brother] was supposed to mean—.
(E) The teacher asked if [the silly story about **Greg’s** older brother] was supposed to mean anything.

Meanwhile, the filled gap effect was found in matched sentences where islands did not prevent a gap as the complement of “about”—reading times at “Greg’s older brother” were higher in the non-island sentence D’ than in the matched if-clause E’. These results show that the active filler strategy applies to the complement of a preposition, as long as this position is not ruled out by a strong island [11].

(D’) The teacher asked what the team laughed about Greg’s older brother fumbling.
(E’) The teacher asked if the team laughed about Greg’s older brother fumbling the ball.

Similarly, a study that investigated the effect of plausibility in strong island vs. non-island sentence structures revealed a plausibility effect in a non-island sentence structure (as in F) on the main verb of a relative clause (*wrote*), such that when the unbounded dependency led to an implausible interpretation (i.e., the city that the author wrote), reading times were longer than when it led to a plausible interpretation (i.e. the book that the author wrote) [13]. However, the same plausibility effect was not observed when the verb was embedded in a strong relative clause island (as in G).

(F) We like the (book/city) that the author **wrote** unceasingly and with great dedication about while waiting for a contract.
(G) We like the (book/city) that the author [who **wrote** unceasingly and with great dedication] saw while waiting for a contract.

Assuming that the access to the relevant plausibility information depends on the parser forming the unbounded dependency, this suggests that the dependency is formed immediately in (F), where there is no island, but that it is not formed in (G), where there is a strong relative clause island. Again, this is consistent with the real-time sensitivity of the processor to island domains, and is compatible with the claim that active dependency formation occurs in non-islands as in (F), but is suppressed in strong islands as in (G).

The studies mentioned above are consistent with the idea that strong island constraints are applied in real time, at the relevant point during the processing of the sentence. Strong islands immediately modulate the parser’s expectancy of a gap [11], or its evaluation of plausibility [13], although there is evidence that active dependency formation is attempted in subject islands incorporating infinitival complements, where a later parasitic gap is possible [19]. Given this, it is important to ask how the processor recognizes the conditions that define an island domain. Previous findings relating to strong islands suggest that one such type of information may be syntactic configuration—for example, if the processor recognizes the left boundary of a constituent that is in the configurational position of an adjunct, such as a relative clause, as in example A.c. above, then it may avoid the expectancy that an unbounded dependency will be resolved inside that constituent. This might be due to a preference to avoid forming the dependency, or perhaps it may even be impossible for the parser to form the dependency at all, due to formal limitations on the range of possibilities allowed by grammatical composition: for example, Steedman [20] points out that the formal operations of Combinatory Categorial Grammar do not allow extraction out of post-modifiers such as relative clauses, as long as standard assumptions are made about syntactic categories. Specifically, extraction from noun modifiers such as relative clauses is impossible in the formalism, assuming that the standard syntactic category (N) is assigned to the modified noun. However, if the noun is allowed to undergo type-raising to receive the complex category N/(N\N), then extraction can in fact occur using the standard composition operations (see [20] p.44. for details). As another example, Marcus [21] proposed a parser that was not able to form unbounded dependencies into relative clauses, due to the way that locality was implemented in the model. The result is that the restrictions on forming dependencies into these types of strong islands could simply be because the necessary formal operations are not available within the grammar or the parser.

Of the studies that have examined the real time processing of islands, the vast majority have tested strong islands. However, it is also important to examine weak islands, as doing so will help us to understand the types of information that the processor uses in order to decide how to process unbounded dependencies in a given type of constituent. As mentioned above, in comparison with strong islands, weak islands often lack clear configurational characteristics in the way that they combine with neighboring constituents (for example, as a subject or an adjunct, in the case of strong islands) that distinguish them from sentences where extraction is possible (recall examples A.a-b, above). Thus, if real-time processing is sensitive to purely configurational relations, then processing might be expected to proceed similarly in A.a and A.b, and active dependency formation would be sensitive to strong, but not weak islands. In contrast, if the information informing this decision also includes features (such as the *wh-*feature of the *whether clause* that distinguishes A.b. from A.a. above), then we would expect real-time dependency formation to be sensitive to both strong and weak islands.

As we mentioned above, few studies have attempted to investigate whether weak islands modulate unbounded dependency formation in real time, though there are studies that have examined the on-line processing of weak islands in the context of memory encoding and retrieval [22, 23]. However, a recent study reported by Villata and colleagues [24], using acceptability judgment and maze-based self-paced reading, suggests that weak islands may modulate active dependency search in a similar way to strong islands. Among the island types that they investigated were both weak islands (*whether-*islands) and strong islands (adjunct islands, complex NP islands). In their maze task, participants had to read a sentence word-by-word, at each stage choosing one out of two words that would form a coherent continuation of the sentence. In critical trials, they recorded whether participants chose a word that was compatible with a gap (e.g. *before* in (H) below), or with the onset of a lexical noun phrase in the place of a gap, (e.g. *the* in (H) below):

(H) What do you wonder whether the candidate solved *the*/*before*…

The continuation-choice results of the maze task showed an effect of islandhood for both weak (*whether*) islands as in (H) and for strong (complex NP) islands: gap continuations were attenuated in both types of islands relative to appropriate baselines. This suggests that both strong and weak islands modulate active dependency formation, since in both cases the expectation for a gap is reduced in the island context. However, these results come from a task that involves a meta-linguistic judgment (choosing one out of two words as a continuation), and the experiments that we report below will consider a similar question in the context of a more natural reading task.

In addition to the question of whether real-time unbounded dependency formation is modulated by both strong and weak islands, the studies that we report below also consider the potential role of *resumptive pronouns* in the processing of long-distance dependencies. There is currently a debate in the literature about whether resumptive pronouns facilitate processing, and particularly, whether such facilitation is modulated by islands, as we will discuss below.

Resumptive pronouns are pronouns that appear in a position where a gap would normally be expected in a filler-gap dependency. To anticipate the experiments reported below, we will illustrate this with two examples from our experiments (I.a is from our Experiment 1 and I.b is from our Experiment 2):

I.a. Jane liked **the magazine** that the hairdresser had talked about **it** before going to the salon.
I.b. This is the magazine that Jane said that the hairdresser had talked about **it** before going to the salon.

In both I.a and I.b, the pronoun *it* appears in a position where a gap would normally be expected. Although resumptive pronouns are grammatical in many languages, they are usually regarded as ungrammatical in English. Given the prior findings on active dependency formation that we discussed above, we would therefore expect *it* to cause processing difficulty in a sentence such as I.a,b, relative to an appropriate baseline condition. This is because *it* would be at odds with the processor’s active expectation of a gap in this position in the sentence, leading to difficulty that is analogous to the filled-gap effect discussed above.

In contrast to the processing difficulty that would be expected on the basis of active dependency formation, there are claims in the literature that resumptive pronouns, such as *it* in (I), *facilitate* processing. In fact, Hofmeister and Norcliffe [25] found that readers speed up following a resumptive pronoun, in sentences like J.b:

(J.a) The prison officials had acknowledged that there was a prisoner that the guard helped to make a daring escape.
(J.b) The prison officials had acknowledged that there was a prisoner that the guard helped him to make a daring escape.

Specifically, the authors reported faster reading times for the two underlined words “to make” in (J.b) (where these words follow the resumptive pronoun *him*) than in (J.a), where the words follow a gap. They argued that resumptive pronouns, despite being ungrammatical, can aid processing in situations where a dependency is difficult to resolve due to demands on cognitive resources (such as when multiple clause boundaries intervene between the filler and the pronoun, as in J.b). This “reverse filled gap effect” in reading time has been recently been replicated by Morgan and colleagues [26], although these authors argue against the claim that the speed-up for resumptive pronouns is indicative of a facilitation of semantic interpretation. Note however, that the reading time facilitation effect is at odds with what would be expected on the basis of active dependency formation, and, to anticipate the studies reported below, it is also at odds with the results reported in this paper. We will return to discuss this matter in the general discussion.

One question that is currently the subject of debate is whether there is a facilitative effect of resumptive pronouns in comprehension that is specific to islands. Studies of English speech corpora and production show that when resumptive pronouns are used, this is mainly in island contexts [1, 27–30].

Several theoretical studies have proposed possible reasons why resumptive pronouns often occur in island contexts [1, 31–33]. For instance, the resumptive pronouns in (K) and (L) occur inside island domains (marked with brackets in the examples below), and are assumed to have a “saving/repair” or “last-resort” function that preserves the grammaticality of the dependency. Therefore, they may intuitively appear to be more acceptable in (K) and (L) than the corresponding gaps in (M) and (N), where the use of gaps is illicit due to the island violation [34].

(K) “I had some other point which I can’t remember [what **it** is].” ([27], p.1554)
(L) “That asshole X, who I loathe and despise [the ground **he** walks on], pointed out that” ([30], p.2)
(M) *I had some other point which I can’t remember [what -—is].”
(N) *That asshole X, who I loathe and despise [the ground **-—**walks on], pointed out that.

The observations above are consistent with evidence from sentence production, showing that the probability of using a resumptive pronoun in an island domain is inversely proportional to the acceptability of the corresponding sentence containing a gap [29], and that speakers have a tendency to “rescue” islands by inserting a resumptive pronoun [35]. This may be because resumptive pronouns are a cognitive strategy to manage the memory burden [36] or it may be due to the incremental nature of production, which can yield locally licensed but globally ungrammatical structures [29, 37].

Despite the abundant evidence regarding the facilitative role of resumptive pronouns in the production of island sentences, there is much less evidence that resumptive pronouns facilitate the *comprehension* of islands. Acceptability-rating experiments have mostly not found that readers judge resumptive pronouns to be more acceptable than the corresponding gaps [3, 32, 38–40], even in island contexts [3, 41], although *comprehensibility* ratings of island sentences with resumptive pronouns have been reported to be increased, relative to the same island sentences with gaps [38] (note that the situation is different in languages where resumptive pronouns are grammatical. For example, in Hebrew, which allows resumptive pronouns in certain environments, people actively search for resumptive pronouns inside relative clauses, where resumptive pronouns are grammatical, but a gap would be ungrammatical [42]). Similarly, participants tended to choose resumptive pronouns over gaps in island contexts given a forced choice of resumptive pronoun or gap (with the expected preference for gaps in non-island contexts) [43].

However, even though resumptive pronouns may show facilitation in some measures, a recent study Morgan et al. [26] has shown, across four experiments, that resumptive pronouns do not facilitate the interpretation of the semantic relation between filler and pronoun in long distance dependencies, whether in island contexts or not. In their Experiment 1, the authors used a multiple choice task to measure listeners’ comprehension of (O.a,b,c), where islandhood (non-island vs. weak island vs. strong island) was crossed with resumption (gap vs. resumptive pronoun):

O.a Non-island.
It is Mr. Dino that Mr. Rabbit said that Miss Piggy tickled __/him with a feather.
O.b Weak Island.
It is Mr. Dino that Mr. Rabbit wondered whether Miss Piggy tickled __/him with a feather.
O.c Strong Island.
It is Mr. Dino that Mr. Rabbit slept while Miss Piggy tickled __/him with a feather.

In (O.a-c), the target interpretation of the long distance dependency corresponds to “Miss Piggy tickled Mr. Dino with a feather”. The authors found that the proportions of these target interpretations were higher in the gap conditions than in the resumptive pronoun conditions, which saw increased proportions of the local interpretation (“Miss Piggy tickled Mr. Rabbit with a feather”), and this tendency was exacerbated in the strong island condition, relative to the no-island condition. This suggests that resumptive pronouns do not help comprehenders to identify the filler (e.g. Mr. Dino in O.a-c) as the antecedent of a long distance dependency, and in fact it suggests that “resumptive” pronouns in English may often be interpreted in a non-resumptive way (i.e. by referring to an antecedent that is not part of the long distance dependency), and this tendency is not reduced when the sentence includes an island. Thus, this study does not support the idea that resumptive pronouns play a facilitatory role in the comprehension of the filler-pronoun relationship, either in general, or in the specific case of islands.

Above, we have argued that previous studies leave open the question of how weak island constraints are deployed during real-time processing, and raise further questions about the relation between island constraints and resumptive pronouns. In the two experiments reported below, we use eye-movement recording during reading to examine how islands are processed in English, using a variant of a filled gap design incorporating a resumptive pronoun. In each experiment, conditions that include an unbounded dependency without an island configuration are compared with conditions with an island. Experiment 1 examines strong relative clause islands. Experiment 2 examines weak islands using a *whether* complement clause. In both experiments, we predict that the resumptive pronoun will disrupt processing relative to a gap in the island conditions, due to the violation of an active expectation for a gap, while this effect is predicted to be absent in the island conditions, assuming that the island inhibits the expectancy for a gap. Such a result in Experiment 1 would be consistent with previous studies that have examined strong islands, while the equivalent result in Experiment 2 would extend this to weak islands, showing that both types of islands constrain processing in real time. Additionally, the experiments offer the opportunity to examine recent claims for a facilitative effect of resumptive pronouns.

Ethical approval for this research was granted by University of Edinburgh Psychology Research Ethics Committee (Ref No: 44-1415/2).

## Experiment 1

Experiment 1 was an eye-tracking study using a 2 × 2 within-subject, within-item design, crossing islandhood (*non-island* and strong *island*) with dependency type (*pronoun* and *a gap*), where the pronoun was used to create a *filled gap* condition, as seen in Example (P) below. Note that this design is similar to that of [25, 26], but differs from typical filler-gap studies such as [11], where the critical region consists of a lexical noun phrase in all conditions, and the design manipulates whether or not the sentence includes an unbounded dependency. We return to this point in the General Discussion.

Example P:
(P.a) *Pronoun / non-island*:
Jane liked **the magazine** that the hairdresser had talked about **it** before going to the salon.
(P.b) *Gap / non-island*:
Jane liked **the magazine** that the hairdresser had talked about before going to the salon.
(P.c) *Pronoun / island*:
Jane liked **the magazine** that the hairdresser who had talked about **it** before going to the salon bought.
(P.d) *Gap / island*:
Jane liked **the magazine** that the hairdresser who had talked about before going to the salon bought.

As discussed above, we predicted that if there is an active expectation for a gap, then we should observe difficulty in (P.a) immediately after *it*, relative to the same position in (P.b). This is because *it* disconfirms the initial prediction of a gap, that would be expected if dependency formation is active. In addition, if active dependency formation is sensitive to islandhood, a filled-gap effect should not be observed in (P.c) when *it* appears inside a strong relative-clause island, because a gap would not be predicted in this case. Given this design, we predicted that active dependency formation would lead to an interaction (all other things being equal) between islandhood and pronoun presence, due to the prediction of a filled gap effect in (P.a) vs (P.b), and the absence of this effect in (P.c) vs. (P.d). It should be noted that this prediction is based on the assumption that active dependency formation in sentences like P.a involves the specific expectation of a gap, meaning that processing difficulty will occur if the putative gap position is filled by any overt noun phrase, including a (potentially) resumptive pronoun such as *it*. However, recalling the discussion in the introduction, we note that that there is some evidence in the literature that resumptive pronouns can decrease reading times, even in languages like English, where they are ungrammatical, [25]. If this is the case, then we would expect a *reverse filled gap effect*, with shorter reading times following *it* in P.a and P.c, than the same sentence position in P.b and P.d. Finally, if resumptive pronouns facilitate processing only in the sense of “saving” island violations, then it is also possible that this facilitation effect is limited to the island conditions (P.c and P.d).

### Method

#### Participants

Forty paid native English-speakers aged 21–24 from the University of Edinburgh participated in the experiment. All were unaware of the study’s purpose, and all gave written informed consent.

#### Apparatus

We used an Eyelink 1000 eye-tracker (SR Research Ltd, Ottawa, Canada) in tower-mounted mode, with a chin rest to stabilize each participant’s head.

#### Materials

Forty items were created based on Example P above (experimental stimuli for both experiments are available at https://osf.io/zbkh9). Each item appeared in the four conditions, crossing islandhood (strong island vs. non-island) with dependency type (pronoun vs. gap). Islandhood was manipulated using relative clauses: the island conditions included relative clauses, while the non-island conditions did not. Dependency type was manipulated by including either a pronoun (*it*) or a gap after the relevant preposition (e.g. *about*).

The forty stimuli were distributed into four lists, following a *Latin Square* procedure. In all four lists, each item appeared in only one condition and each condition appeared an equal number of times. Each list was distributed to ten participants. There were 68 fillers and three practice items, all of which were similar in length to the experimental sentences. The following is a filler example:

- The children laughed at the clown who was hilarious but had forgotten his red nose.

The texts were presented on two or three written lines. Each line had between 90 and 100 characters. Critical regions with a pronoun or a gap always appeared near the middle of a line.

#### Procedures

We presented 108 texts in Times New Roman 18 font, in fixed random order, with no experimental items adjacent. To familiarize participants with the experimental procedure, the experiment began with three fillers. While viewing was binocular, only the right eye was tracked. Items appeared on a 19” monitor approximately 70 cm from a participant’s eyes. In order for the experimenter to check the calibration of each participant, the participant fixated on a black square before each item, which indicated the position of the first character of the text. The black square was automatically replaced with the text once a stable fixation had been detected. After reading each item, the participant pressed a button to end the sentence. For 37% of items, a comprehension question then appeared, which the participant answered by pressing a button on the left or right of the button box. Comprehension questions never probed a pronoun or gap.

### Data analysis

Texts were divided into 5 regions, of which three were used for analysis (see Table 1). Below, we report data for the following regions: pronoun/gap region, spillover, and adverbial. It should be noted there is a length difference in the pronoun/gap region between two levels of the dependency type factor (i.e., *about* vs. *about it*), rendering this main effect uninterpretable. However, our prediction is for an interaction, which is not affected by this confounding variable. Moreover, in our laboratory, we have typically not found early effects of syntactic or referential processing difficulty on short function words like pronouns. We therefore expected that the earliest evidence of an interaction would be found in regression path times, on the immediately following region, namely the spill-over region. This region is matched for length in all conditions, as is the following adverbial region.

**Table 1:** Regions (R) in Experiment 1.

Region	Sample stimulus
R2: Pronoun/gap	about (it)
R3: Spill-over	before
R4: Adverbial	going to

Fixations of less than 80, or more than 1200 ms, were excluded from analysis. All participants correctly answered at least 90% of comprehension questions. Since reporting a large number of eye-movement measures might result in a false positive due to family-wise error [44], we selected only two eye-movement measures. As a measure of initial processing, we selected regression path time, which is calculated as the sum of all fixation durations from first entry into the region from the left, until the first exit of the region to the right. Consequently, regression path time reflects fixation behavior that immediately follows the reader’s first inspection of a given region, before subsequent regions of the sentence are inspected. For completeness, we also report total time (i.e., the sum of all fixations in the region), as a general measure of processing, even though this measure is not informative about initial processing. Both regression path time and total time are typically found to be sensitive to syntactic and referential processing difficulty.

In cases where the region was skipped in initial reading (Regression path time), or where the region received no fixations at all (Total Time), the trial was treated as missing data. For each region and measure, linear mixed effects regression (LMER) models were constructed, incorporating all fixed effects and interactions in a single step. Factor labels were transformed into numerical values, and centered prior to analysis, with a mean of 0 and a range of 1. We performed the analyses on log-transformed reading times. Log reading times were analysed because in both experiments they resulted in models with residuals that provided a better approximation to a normal distribution than analyses based on raw reading times, as judged through the use of qqplot() in R. However, in nearly all cases, significance patterns were the same in the two models (cases where significance differ are indicated in the text). All analyses reported below incorporated crossed random intercepts for participants and items. Random slope parameters corresponding to the two experimental factors and their interactions were included in the maximal model for both participants and items. To aid convergence, and to avoid spurious over-estimates of correlations, random correlation parameters were excluded from the model. The resulting maximal model [e.g. (pronoun * clause type + 1||subject)] converged in most cases. In the gap/pronoun region for regression path, and adverbial region for total time, the model failed to converge, and random slope parameters with the least variance were removed, until convergence was achieved. The results include coefficients, standard errors, and t-values for each fixed effect and interaction. A given co-efficient was judged to be significant at α = 0.05 if the absolute t-value exceeded 2 [45]. Data, scripts and stimuli for both experiments are available at https://osf.io/zbkh9.

### Results

**Fig 1:**
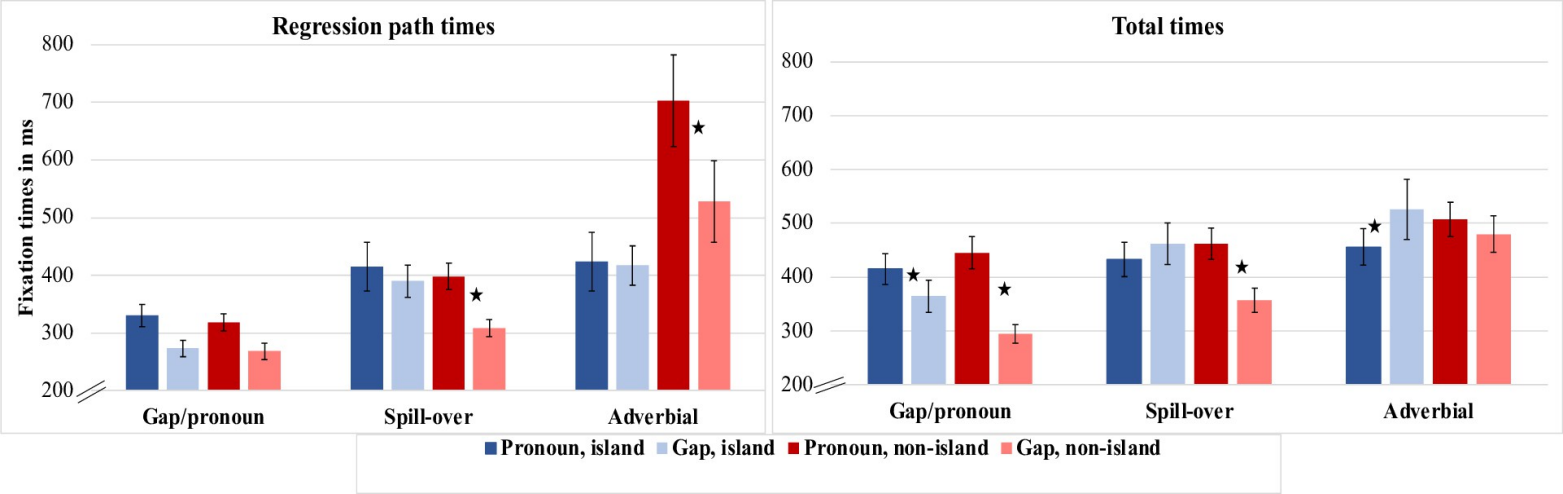
Regression path time and total time (ms) across regions, for Experiment 1.

Means and standard errors for the eye-movement measures are reported in Table 1 (see also Fig 1). Outcomes of statistical tests are reported in Table 2.

**Table 2:** Means (and standard errors), in milliseconds, for regression-path times and total time for Experiment 1.

	Gap/pronoun	Spillover	Adverbial
Regression path times	*M* (*SE*)	*M* (*SE*)	*M* (*SE*)
Pronoun, non-island	318 (15)	398 (23)	703 (80)
Gap, non-island	268 (14)	308 (15)	528 (71)
Pronoun, island	330 (20)	415 (42)	424 (51)
Gap, island	273 (14)	390 (28)	417 (34)
Total time			
Pronoun, non-island	445 (30)	462 (29)	507 (32)
Gap, non-island	295 (17)	356 (23)	480 (34)
Pronoun, island	415 (29)	433 (32)	456 (34)
Gap, island	365 (30)	462 (38)	526 (55)

#### Gap/pronoun region

Regression path and total time showed a main effect of dependency type, with longer reading times for the pronoun condition than for the gap condition: regression path times, Pronoun: *M* = 324 ms, *SE* = 12, Gap: *M* = 270 ms, *SE* = 10; total time, Pronoun: *M* = 430 ms, *SE* = 21, Gap: *M* = 330 ms, *SE* = 18 (see Tables 2 & 3). This effect is likely due to the length differences, as discussed above (e.g., about **it** vs. about).

**Table 3:** Results of mixed-effects analysis for the eye-movement measures for Experiment 1.

	Eye-movement measures					
Regions/parameters	Regression path times			Total time		
	β	*SE*	*t*	Β	*SE*	*t*
Gap/pronoun						
Intercept	5.593	0.029	192.21	5.761	0.047	120.637
DT^a^	0.160	0.032	4.965*	0.238	0.041	5.787*
Islandhood	-0.016	0.034	-0.486	-0.054	0.029	-1.852
Islandhood x DT	-0.023	0.056	-0.421	0.214	0.071	3.006*
Spill-over						
Intercept	5.715	0.038	147.570	5.860	0.011	114.640
DT	0.075	0.037	2.032*	0.076	0.034	2.202*
Islandhood	- 0.051	0.031	-1.649	-0.064	0.034	-1.860
Islandhood x DT	0.159	0.073	2.160*	0.289	0.058	4.926*
Adverbial						
Intercept	5.868	0.046	127.465	5.985	0.061	97.401
DT	0.071	0.047	1.505	-0.031	0.033	-0.937
Islandhood	0.262	0.053	4.878*	0.055	0.026	2.057*
Islandhood x DT	0.268	0.089	3.000*	0.168	0.056	2.995*

^a^DT refers to dependency type.

*Note*. Statistically significant effects are indicated with asterisk (*). Effects are considered significant when the absolute value of *t* was 2 or greater.

Regression path times for the gap/pronoun region did not show a two-way interaction (i.e., islandhood and dependency type). However, in the same region, a significant interaction between the two variables was seen in total time (see Tables 2 & 3 and Fig 1). Within the island conditions, a gap led to shorter total times than a pronoun (*β* = 0.135, *SE* = 0.049, *t* = 2.724). Within the non-island conditions, a gap also led to shorter reading times than a pronoun. This contrast was also significant, but of a much greater magnitude (*β* = 0.346, *SE* = 0.57, *t* = 6.12. It should be noted that both of these pairwise comparisons are affected by the length difference between conditions. Given that there was no evidence for the interaction in regression path time, it is likely that the interaction in Total Time is affected by regressions into the gap/pronoun region from downstream portions of the sentence.

#### Spill-over region

Regression path and total time revealed an interaction between the two variables (Tables 2 & 3 and Fig 1). In the analysis of raw reading times in the spill-over region, the interaction for Regression Path time failed to reach significance, but the main effect of Islandhood was significant for the same measure. Apart from these differences all patterns of significance were the same for the raw and log reading time analyses for Experiment 1. The interaction was due to longer reading times for a pronoun than for a gap in the non-island conditions (regression path: *β* = 0.159, *SE* = 0.040, *t* = 3.923; total time: *β* = 0.218, *SE* = 0.044, *t* = 4.897), but no significant differences in the island conditions (regression path: *β* = -0.005, *SE* = 0.064, *t* = -0.079; total time: *β* = -0.072, *SE* = 0.046, *t* = -1.555).

#### Adverbial region

In regression path times, there was a main effect of islandhood. Reading times were longer in the non-island sentence structure. Both regression path and total time also showed a significant interaction between the two variables. Within the non-island conditions, the pronoun condition had longer regression path and total times than the gap condition. This pairwise comparison was significant in regression path but not in total time (regression path: *β* = 0.203, *SE* = 0.068, *t* = 2.969; total time: *β* = 0.051, *SE* = 0.045, *t* = 1.133). Within the island conditions, there was no significant difference between conditions for regression path (*β* = -0.07, *SE* = 0.051, *t* = -1.344), but for total time, there were shorter reading times for the pronoun condition than for the gap condition (*β* = -0.113, *SE* = 0.044, *t* = -2.60).

### Discussion

In summary, a significant interaction between islandhood and dependency type was observed in both regression path time (i.e., spill-over & adverbial regions) and total time (i.e., gap/pronoun, spill-over & adverbial regions). These findings confirm our predictions based on active dependency formation, and its modulation by islands. According to our interpretation, in the non-island conditions, the presence of a pronoun in a position where a gap is expected in leads to processing difficulty in (P.a), while this difficulty is absent or reduced in the equivalent strong island condition (P.b), presumably because a gap was not expected to occur inside the island. In contrast, the results provide no support for a general facilitative effect of resumptive pronouns on reading times. In particular, in contrast to the results reported by [25], there was no measure in the non-island conditions in which reading times for (P.a) were shorter than those for (P.b). The only evidence for a facilitative effect of the pronoun was in the island conditions, in a relatively late measure of processing. Specifically, in Total Time in the adverbial region, there was an interaction such that, in the island conditions reading times were shorter following a pronoun than a gap (P.c<P.d), but no such difference was observed for the non-island conditions. However, this could simply reflect a grammaticality effect. Consider the relevant conditions, repeated below:

(P.c) Pronoun / island:
Jane liked **the magazine** that the hairdresser who had talked about **it** before going to the salon bought.
(P.d) *Gap / island*:
Jane liked **the magazine** that the hairdresser who had talked about before going to the salon bought.

In (P.c), the sentence continues in a way that eventually allows an unbounded dependency to be formed between *the magazine* and the direct object gap of *bought*. The existence of this alternative gap position means that the pronoun *it* can be interpreted referentially, and the sentence is grammatical. In (P.d), in contrast, the sentence remains ungrammatical. Recall that the Total Time measure can include re-fixations in the region of interest that are made after the reader has previously exited to the right of the region, and can thus reflect processing after the whole of the sentence has been read, allowing an effect of the global grammaticality difference between P.c and P.d. Therefore, shorter total reading times for P.c than for P.d at the word *going* do not necessarily imply a facilitative effect of a resumptive pronoun in an island, but may instead reflect a difference in global grammaticality.

For a related reason, another potential criticism of Experiment 1 is that readers may have predicted the late alternative gap before beginning to process the relative clause island. As the alternative gap position was only available in the island conditions, the prediction of a later gap might have led participants to suspend active dependency formation for the islands, but not for the non-islands. In Experiment 2, we provide a design that does not allow an alternative gap position in the island conditions, and thus removes both of these potential confounds. Another potential criticism is that our use of ungrammatical conditions may have led to the development of artificial strategies over the course of the experiment. However, supplementary analyses of both experiments, including (centered) trial position as an additional factor, failed to show evidence that our critical interaction was significantly modulated by trial position. Thus, the critical experimental outcome does not appear to have changed over the course of the experiment, as might have been expected if participants were developing such strategies.

## Experiment 2

The results of Experiment 1 established real-time sensitivity to island constraints, suggesting that strong islands modulate active gap-filling. This provides a conceptual replication of previous studies discussed above.

In Experiment 2, we asked whether a similar pattern of results could be found for weak islands. The design was similar to Experiment 1, except that the non-island context used a *that* declarative complement clause, and this was contrasted with a weak island context using a *whether* interrogative clause (see sample stimuli in Example Q.).

Example 11:
(Q.a) *Pronoun / that complement clause*.
This is the magazine that Jane said that the hairdresser had talked about it before going to the salon.
(Q.b) *Gap / that complement clause*.
This is the magazine that Jane said that the hairdresser had talked about before going to the salon.
(Q.c) *Pronoun / weak island*.
This is the magazine that Jane wondered whether the hairdresser had talked about it before going to the salon.
(Q.d) *Gap / weak island*.
This is the magazine that Jane wondered whether the hairdresser had talked about before going to the salon.

Each item was matched to the corresponding item in Experiment 1, such that the critical, spill-over and adverbial regions were identical, introducing the same set of discourse referents before the critical region (e.g. “the magazine”, “Jane”, “the hairdresser”).

The use of the *whether* clause allows a better matched comparison between the island and non-island conditions than was possible in Experiment 1. For example, using the *whether* clause manipulation, the number of clause boundaries intervening between the filler and the gap (or pronoun) does not differ as a function of islandhood, which was not the case with the relative clause islands in Experiment 1. Recalling our discussion of weak islands in the introduction, this allows us to examine the sources of information that affect the processor’s expectation of a gap in a filler-gap sentence. In contrast to Experiment 1, where the island conditions had specific configurational properties, in Experiment 2, the island conditions differed from the non-island conditions in features (i.e. the presence or absence of a *wh* feature). In addition, the design does not allow for an alternative gap position in the pronoun conditions, so that in both Q.a and Q.c, no globally grammatical analysis of the sentence is possible. Thus, unlike in Experiment 1, any facilitative effect of the pronoun in the island conditions cannot be explained in terms of a grammaticality effect. Also, unlike in Experiment 1, the island conditions do not allow the possibility to predict a later gap outside the island, making these conditions more comparable with the non-island conditions.

Given the hypothesis that active dependency formation is sensitive to information such as *wh-*features, we predicted an interaction of islandhood with dependency type, as in Experiment 1. In the that-clause conditions (Q.a, b), there is no island, so active dependency formation should result in an expectation for a gap. This expectation is violated when a pronoun appears in the position where the gap is expected, so this should lead to processing difficulty in the regions following the pronoun, with longer reading times in (Q.a) relative to (Q.b), where the expected gap appears. This difference should not be observed in the island conditions (Qc, d), on the assumption that readers do not actively form a dependency inside a *whether* clause, due to its island status. As in Experiment 1, we expected the earliest evidence of this interaction to be established in the spill-over region in regression path time, while the gap/pronoun region should show a main effect of pronoun presence, due to the length difference.

### Method

#### Participants

There were 40 participants in Experiment 2. None of them had participated in Experiment 1. All participants were unaware of the aims of the experiment, and all gave written informed consent.

#### Apparatus

We used the same Eyelink 1000 eye-tracker as in Experiment 1.

#### Materials

The design of Experiment 2 was similar to Experiment 1. Islandhood was manipulated using *that* clauses (non-island) and *whether* clauses (weak island). *It* was always used as the pronoun, as in Experiment 1. Forty items were created by adapting the items from Experiment 1.

#### Procedure

The procedure was the same as Experiment 1.

### Data analysis

Eye movement data and regions were prepared for analysis as in Experiment 1. The analysis was identical in all relevant respects to that of Experiment 1. Again, random correlation parameters were excluded from the LMEs, but remaining random slope parameters were included. If the model failed to converge, random slope parameters with the lowest variance were removed.

### Results

**Fig 2:**
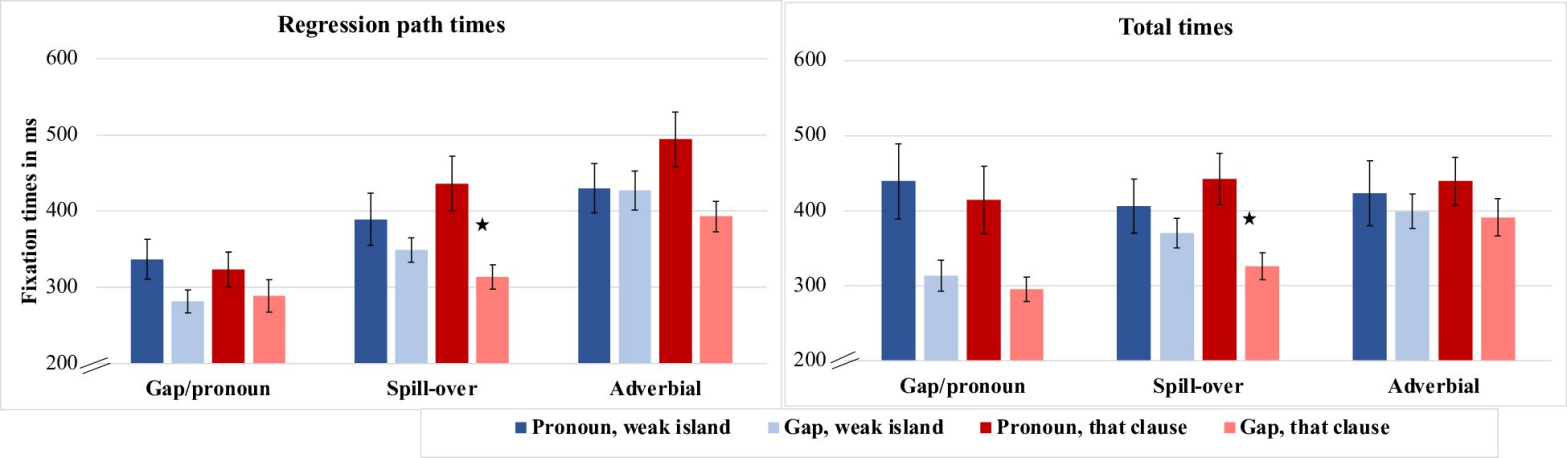
Regression path times and total times (ms) across regions for Experiment 2.

Means and standard errors are given in Table 4 and Fig 2. Outcomes of the statistical tests are given in Table 5.

**Table 4:** Means (and standard errors), in milliseconds, for regression path times and total time for Experiment 2.

	Gap/pronoun	Spillover	Adverbial
Regression path time	M (SE)	M (SE)	M (SE)
Pro, that clause	323 (23)	436 (36)	494 (36)
Gap, that clause	289 (21)	314 (16)	393 (20)
Pro, weak island	337 (26)	389 (34)	430 (32)
Gap, weak island	282 (15)	349 (16)	427 (26)
Total time			
Pro, that clause	414 (45)	442 (34)	439 (32)
Gap, that clause	295 (16)	326 (18)	391 (25)
Pro, weak island	439 (50)	406 (36)	423 (43)
Gap, weak island	313 (21)	370 (20)	399 (23)

**Table 5:** Results of mixed-effects analysis for the eye-movement measures for Experiment 2.

	Eye-movement measures					
Regions/ Parameters	Regression path time			Total time		
	β	*SE*	*t*	β	*SE*	*t*
Gap/Pronoun						
Intercept	5.594	0.041	136.257	5.690	0.581	96.606
DT^a^	0.133	0.039	3.413*	0.247	0.039	6.205*
Islandhood	0.005	0.028	0.191	0.049	0.031	1.560
Islandhood x DT	0.005	0.062	0.086	-0.029	0.064	-0.453
Spill-over						
Intercept	5.708	0.047	120.97	5.764	0.050	115.269
DT	0.106	0.035	2.971*	0.140	0.033	4.194*
Islandhood	-0.028	0.029	-0.959	0.006	0.033	0.189
Islandhood x DT	-0.184	0.058	-3.141*	-0.176	0.054	-3.246*
Adverbial						
Intercept	5.768	0.061	93.584	5.781	0.065	87.994
DT	0.043	0.034	1.271	0.034	0.026	1.289
Islandhood	-0.050	0.032	-1.547	-0.035	0.033	-1.067
Islandhood x DT	-0.134	0.079	-1.694	-0.075	0.064	-1.171

^a^DT refers to dependency type.

*Note*. Statistically significant effects are indicated with asterisk (*). Effects are considered significant when the absolute value of t was 2 or greater.

#### Gap/pronoun region

Both regression path and total time revealed only a main effect of dependency type, with longer reading times for the pronoun conditions than for the gap conditions (regression path: Pronoun: *M* = 330 ms, *SE* = 18, Gap: *M* = 285 ms, *SE* = 13; total time: Pronoun: *M* = 427 ms, *SE* = 33, Gap: M = 305 ms, *SE* = 14. (See Tables 4 & 5 and Fig 2). As in the equivalent region for Experiment 1, this is likely to be due to the length differences of conditions with and without a pronoun.

#### Spill-over region

In regression path and total times, there was a significant main effect of dependency type. Longer reading times for a pronoun than a gap were seen in both eye-movement measures; regression path times: Pronoun: *M* = 412 ms, *SE* = 25, Gap: *M* = 331 ms, *SE* = 11; total times: Pronoun: *M* = 427 ms, *SE* = 33, Gap: M = 304 ms, *SE* = 14. In both eye-movement measures, there was also a significant two-way interaction between islandhood and dependency type (see Table 4). Among the *that-*clause conditions, there were longer regression path and total times for the pronoun conditions than for the gap conditions, and this was significant in the pairwise comparisons (regression path: *β* = 0.2, *SE* = 0.047, *t* = 4.229; total time: *β* = 0.228, *SE* = 0.040, *t* = 5.655). In the island conditions there were no significant reading differences between pronoun and gap conditions (regression path: *β* = 0.024, *SE* = 0.045 *t* = 0.536; total time: *β* = 0.057, *SE* = 0.044, *t* = 1.282).

#### Adverbial region

Neither the main effects nor the interaction reached significance for either Total Time or Regression Path Time. In the analysis of raw reading time, the main effect of Dependency Type was significant for Regression Path. Apart from this difference, all significance tests yielded the same outcome for raw and log reading times for Experiment 2.

### Discussion

The results of Experiment 2 are consistent with the idea that active dependency formation is sensitive to weak islands. When the sentence incorporated a *that* clause, readers slowed down following a pronoun, relative to when the sentence contained a gap. As the that-clause is not an island, our interpretation of this affect is that readers predicted a gap, and the subsequent appearance of a pronoun in the expected gap position led to processing difficulty. In contrast, when the sentence incorporated a *whether*-clause, processing difficulty following the pronoun was attenuated. The resulting interaction was observed in both Regression Path Time and Total Time in the spill-over region. We interpret this pattern of results to show that active dependency formation is sensitive to weak islands, just as it is sensitive to strong islands, as suggested by the results of Experiment 1.

Experiment 2 showed no evidence for any facilitative effect of resumptive pronouns on reading times. For the non-island conditions, there was no measure in which reading times for the pronoun condition were significantly shorter than those of the gap condition. This lack of facilitation is consistent with Experiment 1, but runs counter to the results reported in [25]. For the island conditions, there was also no observable reading time facilitation for the pronoun relative to the gap. This suggests that the speed-up for the pronoun condition in Experiment 1 in Total Time in the adverb region may have been spurious, or due to a grammaticality confound as discussed above.

## General discussion

Previous studies on active-dependency formation have shown that readers integrate their grammatical knowledge of strong islands in real time, guiding expectations about completing a filler-gap dependency [11, 13, 14] (see also [19] and references cited therein). Our results support these findings, showing the real-time sensitivity to both strong and weak islands. We interpret this to mean that active dependency search depends on the island status of the relevant constituent, and that this applies not only to a strong island (Experiment 1), but also to a weak island configuration (Experiment 2). The (strong) relative clause subject island that we used in Experiment 1 provides a very clear structural cue to the processor, with the relative pronoun indicating an extra level of embedding (*Jane liked the magazine that the hairdresser* ***who*** *had…*), relative to the non-island condition (*Jane liked the magazine that the hairdresser had…*). In contrast, the *whether* island used in Experiment 2 differed from its non-island condition in lexical content (and consequently in syntactic features) but the two conditions were closely matched structurally, with no difference in the levels of embedding (*This is the magazine that Jane wondered whether the hairdresser had…*) vs. (*This is the magazine that Jane said that the hairdresser had…*). This suggests that the decision of whether to form an active expectancy for a gap within a given constituent takes into account not only aspects of structural form (such as levels and types of embedding), but also feature-based content (such as whether a clause has a *wh* feature).

Given that we have found similar results for both strong and weak islands, one important question is whether strong and weak islands differ at all in processing. On the basis of off-line acceptability ratings, as well as continuation-choice data from a maze task, Villata and colleagues [24] argued against a categorical distinction between weak and strong islands, arguing instead that the propensity to posit a gap in a given constituent is negatively correlated with the severity of the island violation (n.b., we discussed this study in the introduction, in relation to the finding that gap-continuations were attenuated in both strong and weak islands: see discussion around example H). In addition, for both weak (*whether*) islands as and strong (complex NP) islands, gap continuations were chosen more often in conditions with a complex initial *wh* phrase (e.g. *Which problem do you wonder whether the candidate solved the/before…*) relative to a simple *wh* phrase (e.g. *what do you wonder whether the candidate solved the/before*), a manipulation that has traditionally been assumed to affect the acceptability of only weak islands. This pattern correlated with preferences from an offline forced choice task, where for both types of island, complex *wh*-phrase sentences were rated as more acceptable than their simple *wh-*phrase counterparts—this can be interpreted as further evidence for the lack of a distinction between strong and weak islands, because the traditional view is that only weak islands (and not strong islands) are improved by using complex wh-phrases. Thus, it might be the case that active gap filling for both strong and weak islands may not depend on an *all-or-nothing* decision, but may instead be a matter of degree. According to such an account, the decision of whether to predict a gap in an island constituent might vary stochastically, with the probability of predicting the gap correlating negatively with the severity of the island, and the highest probability for non-islands. In terms of the experimental designs of the studies we report here, such an account would predict an interaction of the same form that we report above, with a greater cost for the pronoun (relative to the gap) in the non-island conditions than in the island conditions. However, the account would also predict that such a pronoun cost should also be detectable even in the island conditions, given a study with sufficient power, and that the magnitude of this effect would be greater for weak islands (which are typically judged as less severe) than for strong islands. Although our Experiment 2, which examined weak islands, yielded no measure in which both the two-way interaction and the pairwise comparison as described above were significant, we do not rule out that a study with greater power would do so.

Our experimental predictions were based on the assumption that the critical pronoun in our stimuli would play the role of a filled gap, leading to an increase in the non-island condition, as was seen in the results across two experiments. However, as mentioned above, other recent studies have shown that, rather than increasing reading times, a pronoun in the position of a predicted gap can *decrease* reading times. In the following paragraphs, we review this evidence, and offer some speculations about why the results may have differed.

Recall from the introduction to this paper that a study by Hofmeister and Norcliffe [25] found decreased reading times for H.a relative to H.b; we repeat two corresponding conditions from our own study below in Qa and Qb.

H.a. Mary confirmed that there was a prisoner who the prison officials had acknowledged that the guard had helped **to make** a daring escape.
H.b. Mary confirmed that there was a prisoner who the prison officials had acknowledged that the guard had helped him **to make** a daring escape.
Q.a. This is the magazine that Jane said that the hairdresser had talked about **before** going to the salon.
Q.b. This is the magazine that Jane said that the hairdresser had talked about it **before** going to the salon.

Specifically, the previous self-paced reading study [25] found shorter reading times at the underlined region following a pronoun in (H.b) than following a gap in (H.a), while Experiment 2 of the present paper found the opposite result, with longer regression path and total times following a pronoun in (Q.b) than a gap in (Q.a). Thus, while our study supports active dependency formation, the previous study [25] does not, and instead shows a reading time facilitation for the pronoun condition.

To our knowledge, there is one other study, by Morgan and colleagues [29] (discussed in the introduction) that has found shorter reading times following a resumptive pronoun than following a gap. In their Experiment 2 (self-paced reading), these authors manipulated not only pronoun presence, but also islandhood, examining both weak and strong islands, as in R.a-c:

R.a (non-island).
It was Miss Piggy that Miss Cat reported that Mr. Dog poked __/her with a pencil.
R.b. (weak island).
It was Miss Piggy that Miss Cat understood why Mr. Dog poked __/her with a pencil.
R.c. (strong island).
It was Miss Piggy that Miss Cat snacked while Mr. Dog poked __/her with a pencil.

In the non-island condition (R.a), the authors found faster reading times at the second word following the resumptive pronoun (“her”) than in the same position following a gap. Their Bayesian analysis did not provide any evidence that this pronoun effect was modulated by island status, either for the weak or the strong islands, relative to the non-island baseline. Although the authors’ main focus was on the semantic interpretation of the sentence, rather than on the reading times, it is nevertheless striking that (a) there was no evidence for the filled-gap pattern in the non-island condition, which would be predicted by active dependency formation, and (b) that the reading times showed no evidence of interaction between pronoun and islandhood. Both of these aspects of the results differ from those of the two experiments reported in the present paper.

In the following paragraphs, we suggest some potential reasons why the reading time results in the present paper might have differed from those of the two studies described above. The first question we consider is why we found the pronoun cost (relative to gaps) in the non-island conditions, an effect that is indicative of active dependency formation, while the other two studies [25, 26] did not. Here, we will concentrate on our Experiment 2, which used sentences of a similar structure to those of both [25, 26]. In our Experiment 2, all the sentences in our non-island conditions used either *thought* or *said* as the complement-selecting verb, both of which are prototypical *bridge verbs*, defined as verbs that readily allow extraction out of their complements in unbounded dependencies (e.g. *What did you say that John ate*?) [46], as opposed to non-bridge verbs (e.g. *whisper*), where extraction is more awkward (e.g. *What did you whisper that John ate*?). Recent research suggests that, even within verbs that are viewed as “bridge verbs”, there are differences in acceptability of extraction, with extraction being judged most acceptable for *think* and *say* and slightly degraded acceptability for other complement-selecting verbs [47], and it may be the case that the “bridge” status of verbs is better thought of as a gradient, rather than a dichotomy [48]. Although Hofmeister and Norcliffe [25] do not provide a full set of experimental materials, the example that they give uses *acknowledged*, which is not among the most prototypical bridge verbs. As for Morgan and colleagues [26], these authors used 48 items, out of which 12 used prototypical bridge verbs *thought* or *said*, and the remainder used verbs that were less prototypical as bridge verbs (e.g. *replied*, *asserted*, *reported*, *swore*). It may be the case that active dependency formation is optimized for the most prototypical bridge verbs, while less predictive processing strategies may sometimes be used in the context of other verbs, even if extraction using these verbs is perceived to be globally grammatical. If this is correct, the relatively low proportion of prototypical bridge verbs might explain why Morgan and colleagues [26] did not observe a filled-gap-like effect in the non-island conditions in their self-paced reading study, and would also explain why their reading time results did not show an interaction between pronoun presence and island status. According to this view, the use of non-prototypical bridge verbs might have meant that their non-island conditions were effectively treated as (weak) islands in a proportion of trials, from the point of view of active dependency formation, making it harder to detect a modulating effect of islandhood on the pronoun effect in their study. In contrast, both the filled-gap-like effect and the interaction between pronoun presence and islandhood were observed in our Experiment 2, where we exclusively used the prototypical bridge verbs *said* and *thought*, as would be expected if the use of these verbs optimized active dependency formation in the non-island conditions. Of course, our explanation is speculative at this stage, and it would require a series of empirical studies to test the assumptions on which it is based.

The second question that we consider is that of why both Morgan and colleagues [25] and Hofmeister and Norcliffe [26] observed a speed-up for their pronoun conditions relative to gap conditions, even though we observed an opposite effect in our non-island baseline. Morgan and colleagues [26] attribute this effect to a sense of confusion on the part of their participants in the pronoun conditions, presumably regarding the choice of referent for the pronoun in the context of quite a complex sentence, and indeed their comprehension accuracy data showed that the use of resumptive pronouns led to lower rates of accuracy for interpreting the relevant dependency relation than the use of gaps did (see discussion in the introduction above). This “confusion” effect may be related to the ambiguity advantage effect, which has been shown to apply to pronouns [49], such that pronouns with two feature-matching antecedents facilitate processing relative to those with one matching antecedent. Further work would have to be done in order to corroborate this “confusion” effect, and to explain why it did not appear in our studies. We speculate that this may be related to animacy: the resumptive pronouns in our study were all inanimate (*it*), and most items included only one inanimate entity in the sentence context, while the pronouns used by [25, 26] were animate, with more than one animate entity mentioned in the sentence context. If “confusion” is modulated by animacy, then this might explain the difference between our study and the previous studies. Again, this explanation is speculative, and would need to be tested in further empirical work.

Despite the evidence that resumptive pronouns do not facilitate the interpretation of dependencies [26], there is some recent evidence that they can do so, at least in some circumstances, specifically in cases where cognitive resources are particularly taxed. In a series of speeded binary acceptability studies [39], increasing the demand on working memory, either by lengthening the dependency or by adding an extrinsic memory load, increased participants’ acceptance rates of ungrammatical sentences with “gap-less” unbounded dependencies, like S.a and S.b:

S.a. This is the babysitter that the butler said that her friend really liked kids.
S.b. This is the butler that the maid said that her friend really liked kids.

However, in high memory load conditions, S.a was accepted more often than S.b. Under normal conditions, the processing of a filler leads to the prediction of a structure including a gap [39]. However, according to the author this prediction can decay when working memory load is high, and in such cases, a resumptive pronoun can help the reader to form a coherent interpretation of the sentence. Thus, the higher acceptance rate of S.a relative to S.b can be explained as the resumptive pronoun *her* matches the stereotypical gender of the filler *the babysitter* in S.a, but this is not the case in S.b, where *her* mismatches with *the butler*.

Finally, we discuss some limitations of our design. We have argued that both weak and strong islands inhibit the expectancy for a gap to occur within the relevant constituent. This is based on our assumption that the reading time slowdown that we observed for the pronoun, relative to the gap, in the non-island conditions, was due to the violation of the expectation for a gap, and that the lack of such a disruption in the island conditions was due to the lack of such an expectation.

Recall that our design involves a direct comparison of conditions including a pronoun (which could in principle be interpreted as co-referential with the preceding filler phrase) with those including a gap. This type of design is similar to those of other studies that have examined resumptive pronouns [25, 26], but it differs from typical *filled gap* designs, where a condition involving an unbounded dependency and a lexical noun phrase in the place of a predicted gap is contrasted with a control condition that includes the same lexical noun phrase but without an unbounded dependency. Stowe’s study [11], which we discussed above, is an example of the more typical filled gap designs, as it included not only conditions where *filled-gap* processing difficulty was expected (see example D’ from Stowe’s study, repeated below), but also control conditions that did not involve an unbounded dependency (see example E’), and therefore would not be predicted to involve the expectation of a gap. These can be compared with examples Q.a and Q.b from our Experiment 2, repeated below:

(D’) The teacher asked what the team laughed about Greg’s older brother fumbling.
(E’) The teacher asked if the team laughed about Greg’s older brother fumbling the Q
Q.a. This is the magazine that Jane said that the hairdresser had talked about it before going to the salon.
Q.b. This is the magazine that Jane said that the hairdresser had talked about before going to the salon.

We acknowledge that there are disadvantages for the design that we used. For example, in our design, it is not possible to control for differences in n-gram probability around the critical region: the sequence *talked about it* in (Q.a) presumably has a higher probability than *talked about before* in (Q.b). However, our primary prediction was for an interaction, namely, a difference in the size of effect between (Q.a) and (Q.b) relative to the size of effect between the two island conditions, and this interaction is not affected by this n-gram confound. In addition, for the non-island conditions, our prediction is for a reading time cost for the *pronoun* condition, which is the opposite of the prediction of an account based on n-grams. The traditional type of filled-gap design, which does not suffer from the n-gram confound, directly compares sentences with and without extraction dependencies (see D’ and E’), but it is still theoretically possible that the overall difference in structure could affect processing, and therefore reading times, independently of filled gap effects.

A second potential drawback of our design, which is again related to the lack of a non-unbounded dependency control condition, is that the cost for the pronoun (relative to the gap) that we found in the non-island conditions, could be interpreted as an effect of grammaticality, or relative preference, rather than an effect of violation of expectation, due to the ungrammaticality of resumptive pronouns in English, in comparison to the perfectly grammatical gap in the same position. The lack of such a pronoun cost in the island conditions could then be explained if we assume that the processor treats both gap and pronoun as ungrammatical or sub-optimal at this point (the gap is ungrammatical because of the island constraint, and the pronoun is arguably ungrammatical independently of islands because it is resumptive). Note that this account assumes that the processor can detect at this point in the sentence that the pronoun must inevitably be interpreted as an ungrammatical resumptive pronoun, and that the sentence cannot be saved by a gap later, in both island and non-island conditions. Arguably, the argument applies better to the materials of Experiment 2 than Experiment 1, where we believe that the pronoun is interpretable as a referential pronoun (see discussion section of Experiment 1). Overall, then, this explanation could explain the interaction that we observed, without assuming that the processor adopts an expectation for a gap in any of our four conditions, since the pronoun or gap could be identified bottom-up, and the grammaticality of the sentence could be evaluated at this point in the sentence, without an expectation having been set beforehand.

Although we assumed that active dependency formation would apply to our non-island conditions, on the basis of previous studies such as that of Stowe [11], we acknowledge that our design does not rule out the non-predictive account sketched above. However, in order to explain the critical interaction that we observed, we must assume that island constraints were applied in real time during incremental processing of the sentence, whether the dependency was formed predictively or not. According to the active dependency explanation, the expectation for a gap was invoked in the non-island conditions before the gap or pronoun was reached in the input, but this expectation was suppressed in the island conditions, through the top-down invocation of grammatical knowledge, at the point where the island domain was entered. According to the non-predictive explanation, the top-down expectation for a gap was not invoked in any of the four conditions, but grammatical constraints were applied immediately, as soon as bottom-up evidence was received for either a pronoun or a gap, leading to the observed interaction: the slow-down for the pronoun (relative to the gap) in the non-island condition due to the ungrammaticality of resumptive pronouns, and the lack of such a difference in the island conditions due to both conditions being either ungrammatical or sub-optimal. Thus, whichever explanation is adopted, island constraints must have been applied in real time, either during the formation of expectancies, or at the point where pronoun or gap was recognized.

A final question is whether the resumptive pronouns were interpreted as co-referential with the filler phrases in our experiments (for example, whether *it* was interpreted as co-referential with *the magazine* in Q.a). Although the previous study by Morgan et al. [26] (see discussion above) suggests that co-reference with the filler does not invariably occur when multiple alternatives are possible, in our study, the filler is the only plausible antecedent for the pronoun, and this anaphoric link may have been made, despite the ungrammaticality of resumptive pronouns in English. However, we point out that the issue is orthogonal to our main research question, which is whether island domains lead the processor to suspend active expectation for a gap. In principle, once the gap or pronoun has been recognized in the input string, the link between *it* and the filler phrase could be interpreted whether or not there had been a prior expectation for a gap. Moreover, if such an expectation were made, the appearance of the pronoun in the input would be expected to lead to processing difficulty, due to its form mismatch relative to the expected gap, and this could occur independently of whether the anaphoric link is subsequently made. More generally, in principle, the link could be made whether or not grammatical constraints are applied on-line: in other words, interpretation of the pronoun as co-referential with the filler could occur whether or not the dependency has been recognized as grammatical at the relevant point in processing.

## Conclusion

In this paper, we have replicated previous findings that on-line sentence comprehension is sensitive to strong islands (Experiment 1), and we have shown novel evidence that it is also sensitive to weak islands (Experiment 2). We interpret this to mean that the decision of whether to predict a gap is sensitive not only to the configurational information that defines strong islands, but also to the featural information that defines weak islands. Moreover, the results show no strong evidence for a facilitatory effect of resumptive pronouns.
